# A new anatomical insight into the aetiology of lateral trunk of suprascapular nerve neuropathy: isolated infraspinatus atrophy

**DOI:** 10.1007/s00276-018-1996-2

**Published:** 2018-03-09

**Authors:** Anna Fabis-Strobin, Miroslaw Topol, Jaroslaw Fabis, Kryspin Niedzielski, Michal Podgorski, Lukasz Strobin, Michal Polguj

**Affiliations:** 1 Clinic of Orthopaedic and Traumatology, Polish Mother’s Memorial Hospital Research Institute, Rzgowska 281/289, 93-338 Lodz, Poland; 2 Department of Normal and Clinical Anatomy, Interfaculty Chair of Anatomy and Histology, Medical University of Łódź, Narutowicza 60, 90-136 Lodz, Poland; 3 Department of Arthroscopy, Minimally Invasive Surgery and Sports Traumatology, Medical University of Lodz, Zeromskiego 113, 90-549 Lodz, Poland; 4 FMC Medical Center, Pilsudskiego 9a, 90-368 Lodz, Poland; 5 Department of Diagnostic Imaging, Polish Mother’s Memorial Hospital Research Institute, Rzgowska 281/289, 93-338 Lodz, Poland; 6 Faculty of Technical Physics, Information Technology and Applied Mathematics, Lodz University of Technology, Wolczanska 215, 90-924 Lodz, Poland; 7 Department of Angiology, Interfaculty Chair of Anatomy and Histology, Medical University of Łódź, Narutowicza 60, 90-136 Lodz, Poland

**Keywords:** Suprascapular neuropathy, Isolated infraspinatus atrophy, Throwing sports, Suprascapular nerve anatomy

## Abstract

**Introduction:**

Although the pathomechanism of isolated infraspinatus atrophy (ISA) in throwing sports is known to be traction, it is unclear why only some players are affected. One likely explanation is that the infraspinatus pulling force exerted by its contracture generate the compressive resultant component force (Fn) compressing the lateral trunk of the suprascapular nerve (LTSN) against the edge of scapular spine. This paper makes two key assumptions (1) the course of LTSN in relation to the scapular spine, defined as the suprascapular-scapular spine angle (SSSA) is the key individual anatomical feature influencing the Fn magnitude, and thus potentially ISA development (2) SSSA is correlated with scapular notch type.

**Materials and methods:**

The bone landmarks of the LTSN course were identified in 18 formalin-fixed cadaveric shoulders, and the SSSA was measured in 101 dry scapulae. The correlation between the SSSA and suprascapular notch type was evaluated. The Fn value was simulated mathematically based on the values of the SSSA of 101 dry scapulae and the prevalence of ISA in chosen throwing sports, as given in the literature: i.e., beach volleyball − 34% (group A1 − 34%; group A2—remaining 66% of scapulae) and tennis − 52% (group B1 − 52%; group B2—remaining 48% of scapulae).

**Results:**

The mean SSSA value was 44.57° (± 7.9) and Fn 79 N (± 13.1). No statistically significant correlation was revealed between suprascapular notch type and SSSA. Groups A1 and B1 possessed significantly lower SSSA values (*p* < 0.000) and significantly higher Fn magnitude (*p* < 0.000) than groups A2 and B2 respectively. The average difference of Fn was 28.1% between group A1 and A2 and 31% between group B1 and B2.

**Conclusions:**

The SSSA has a wide range of values depending on the individual: the angle influencing the magnitude of the compressive resultant force Fn on the LTSN at the lateral edge of the scapular spine via contraction of the infraspinatus muscle. The prevalence of ISA in throwing sports may be correlated with the SSSA of the LTSN. However, further combined clinical, MRI or/and CT studies are needed to confirm this.

## Introduction

Despite the passage of time, the aetiology of isolated atrophy of the infraspinatus (ISA) in throwing sports remains poorly understood. Its frequency of occurrence can be surprisingly high, reaching 34% in beach volleyball [22], with an average prevalence of 52% among elite female tennis players [50]. It has been suggested that in these cases, its development occurs as a result of nerve traction during repeated overhead motion of the shoulder [4, 12, 17, 21–23, 28, 34, 36, 39, 42, 46, 47, 50], however, it is unclear why only some players are affected. Therefore it seems the pathomechanism must be more complex and associated with the key anatomical player, i.e., the lateral trunk of the suprascapular nerve (LTSN) innervating the infraspinatus [16]. From a biomechanical point of view, the angle between the LTSN and the scapular spine, i.e., the suprascapular-scapular spine angle (SSSA), appears to have a key influence on the magnitude of the resultant compressive force (Fn) acting on the LTSN at the edge of the scapular spine [39] by pulling force (*F*) exerted by contraction of the infraspinatus.

The aim of the present study was to clarify the biomechanical role of LTSN pulling in the development of ISA in throwing sports. To this end, the following secondary aims were pursued: (1) to identify the bone landmarks of the LTSN course in preparations of formalin-fixed cadaveric shoulders, (2) to measure the SSSA in dry scapulae, (3) to evaluate the occurrence of a particular type of scapular notch among the dry scapulae. (4) to calculate the Fn value based on the magnitude of SSSA with regard to the prevalence of ISA in chosen throwing sports, as given in the literature: i.e., beach volleyball − 34% [22] and tennis − 52% [50].

Based on the acquired data, the study verifies the following hypotheses: (1) the course of the LTSN in relation to the scapular spine, defined as the SSSA, is an individual anatomical feature and thus influences the magnitude of the compressive resultant force Fn of the infraspinatus pulling force *F* exerted on it by infraspinatus muscle contraction; (2) the SSSA value depends on the type of scapular notch; (3) the acquired test results represent a valuable step forward to understanding ISA aetiology and act as a basis for further clinical research.

## Materials and methods

The tests were performed in two stages: The first using 18 formalin-fixed, cadaveric shoulders and the second using 101 dry scapulae. All tested preparations, obtained from the collection of the Department of Normal and Clinical Anatomy of Medical University of Lodz, were undamaged, and taken from cadavers of unknown sex and age. The research project was approved by the Bioethics Commission of the Polish Mother’s Memorial Hospital Research Institute (protocol no. 11/2017).

**Fig. 1:**
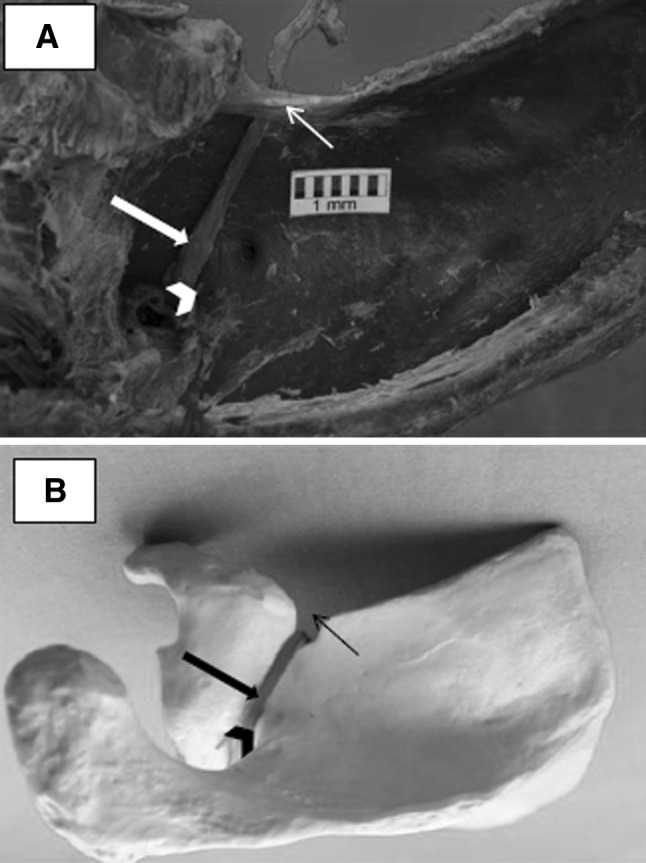
**a, b** The course of the lateral trunk of the suprascapular nerve, confirmed by anatomical examination. **a** Permanent soft preparation of the upper limbs, posterior-superior view of the suprascapular fossa. **b** Dry preparation of the scapula indicating the course of the lateral trunk of the suprascapular nerve. Large arrow—lateral trunk of the suprascapular nerve, small arrow—superior transverse scapular ligament, arrowhead—lateral edge of the scapular spine

**Fig. 2:**
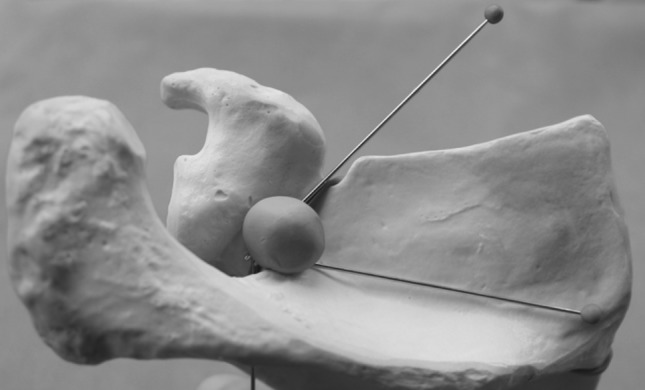
The tool used for measuring the suprascapular spine angle (SSSA), and the means by which the angle was determined with regard to the reference points on the bone (bottom of scapular notch, lateral point of scapular spine edge, medial and lateral points of upper aspect of scapular spine), and the method of preserving the position of the measuring device using plastic materials

The upper limbs were examined to establish the bone landmarks of the LTSN course. In all preparations, a horizontal cut was made along the clavicle, and the skin was dissected from the deltoid, trapezius and pectoralis major. The deltoid and the trapezius were then removed. The upper edge of the scapula and the area around its notch were revealed by dissecting the supraspinatus muscle (Fig. 1). Next, based on the test results from the soft preparations of the upper limbs, the course of the LTSN was determined on the dry scapulae and the SSSA was measured. The SSSA is the angle included between the line running through the lowest point at the base of the scapular notch and the distal point of the scapular spine edge and the line joining the most prominent medial and lateral points of superior aspect of scapular spine. The scapular spine arm of the tool used for SSSA measurement automatically positioned itself on the two most superior points (lateral and medial) of the scapular spine. The arm was set in fixed plastic material, allowing a precise reading to be taken of the angle between the arms of the tool (Fig. 2). The arrangement of the measuring tool arms indicated the course of the LTSN in the fixed preparation.

The SSSA was measured four times in an independent series of measurements performed every 2 weeks. The measurements were performed by the first author AFS (2 measurements) and co-author MP (2 measurements). The inter- and intra-observer reliability tests were done (interclass correlation coefficient). The mean value of the four measurements was used for the statistical analysis.

**Fig. 3:**
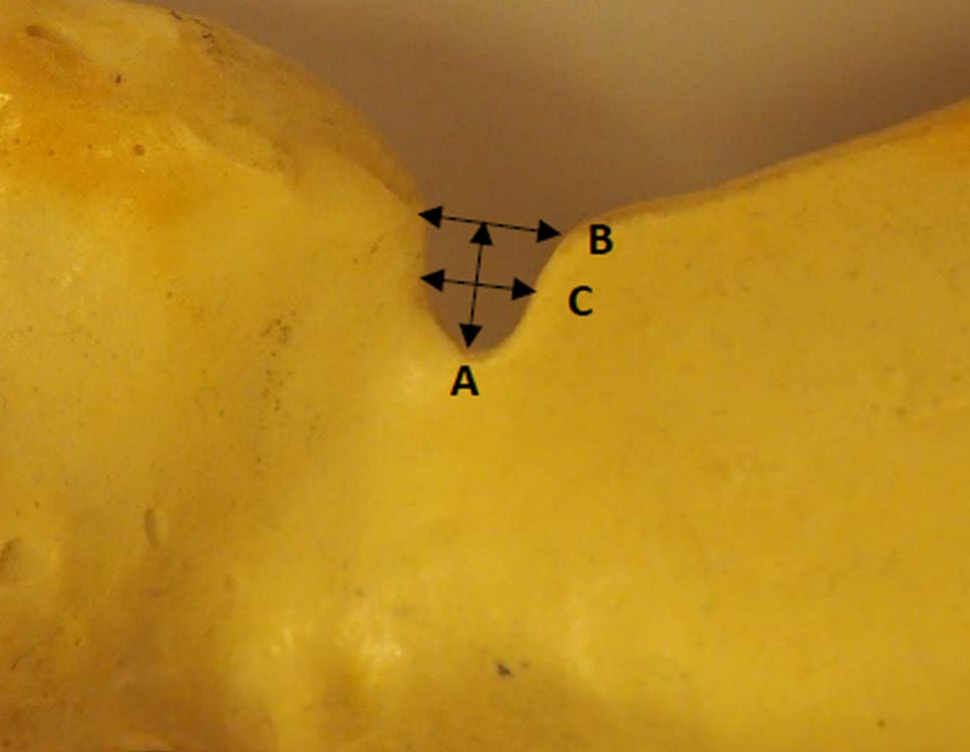
Suprascapular notch dimension measurements of maximal depth **a**; superior transverse **b**; middle transverse **c** according to classification proposed by Polguj et al. [31]

In the second stage, the 101 dry scapular preparations were classified according to notch-type classification based on measurements of its dimensions according to Polguj et al. [31] (Fig. 3; Table 1). All measurements were performed with an analogue calliper with a resolution of 0.05 mm (MITUTOYO, Japan).

**Table 1:** Suprascapular notch classification based on the notch dimension measurements of maximal depth (A); superior transverse (B); middle transverse (C) according to Polguj et al. [31]

Notch type	Classification criteria
I	General *A* > *B* Ia *B* < *C*; Ib *B* = *C*; Ic *B* > *C*
II	*A* = *B* = *C*
III	General *A* < *B* IIIa *B* < *C* ; IIIb *B* = *C* ; IIIc *B* > *C*
IV	Bony foramen
V	Discrete notch

To examine the hypothesis that the SSSA value influences the risk of lateral trunk suprascapular neuropathy (LTSN) four groups were separated from the 101 scapulae under investigation—A1, A2, B1 and B2. Group A1 and B1 were formed to include the proportion of scapulae from the total test sample which reflects the proportion of scapulae demonstrating LTSNN in beach volleyball players (34% of 101 scapulae; *n* = 34) [22], and in tennis players (52% of 101 scapulae; *n* = 52) [50]. In addition, two groups were formed to reflect the proportions of players who do not suffer from LTSNN: Group A2 for the beach volleyball players (*n* = 67 scapulae [101—34]) and Group B2 for the tennis players (*n* = 49 scapulae [101—52]). The statistical analysis examined the comparison between groups A1 and A2 and groups B1 and B2.

**Fig. 4:**
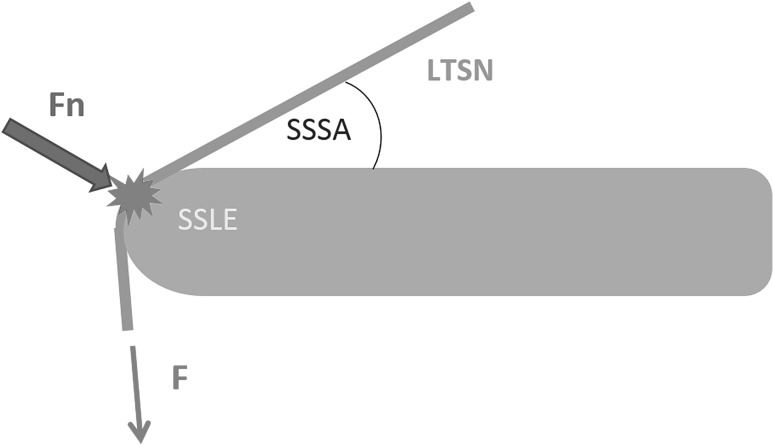
The schematic drawing of resultant compressive force (Fn) of the pulling force of infraspinatus contraction (*F*), acting on the lateral trunk of the suprascapular nerve (LTSN) against the scapular spine lateral edge (SSLE). *SSSA* suprascapular spine angle

The compressive force acting on the LTSN (Fn) against the scapular spine edge is the resultant compressive component of the pulling force exerted by the contraction of the infraspinatus (*F*) (Fig. 4) [13]. To calculate the hypothetical magnitude of Fn of pulling force F of infraspinatus, the appropriate physical formula was used [13]. The magnitude of the infraspinatus pulling force (*F*), i.e. 205 N, was taken from a study by Escamila et al. [8]. Therefore, the hypothetical calculation of force Fn was based on the following three components: (1) The physical formula $\left[ {{\text{Fn}}\;{\text{=}}\;F{\text{sin}}\frac{{\left( {90^\circ ~ - ~{\text{SSSA}}} \right)~~}}{2}} \right],$ derived by taking the force component perpendicular to the edge [13]; (2) the average value of infraspinatus force (*F*) according to Escamila et al. [8] i.e., 205 N; (3) the value of SSSA evaluated in the present study.

### Statistical analysis

A power analysis was performed (*α* = 0.05, 80% power). The effect size (*d*) of the differences were calculated (small if 0 ≤ |*d*| ≤ 0.5, medium if 0.5, |*d*| ≤ 0.8, and large if |*d*| > 0.8). The intra- and inter-observer correlation (interclass correlation coefficient) were calculated for the four measurements of SSSA. The normality of the data was evaluated using the Shapiro–Wilk test. Student’s *t* test was used for normally distributed data with equal variances, and the *U* Mann–Whitney test for non-normally distributed data. *p* values below 0.05 were regarded as statistically significant.

All calculations were performed with STATISTICA ver10. (StatSoft, Inc, Pl, 2011; http://www.statsoft.com) The correlation between the SSSA and suprascapular notch type was calculated using Weka 3: Data Mining Software in Java [15] designed for data mining and detection of complex relationships between the analyzed values.

## Results

**Fig. 5:**
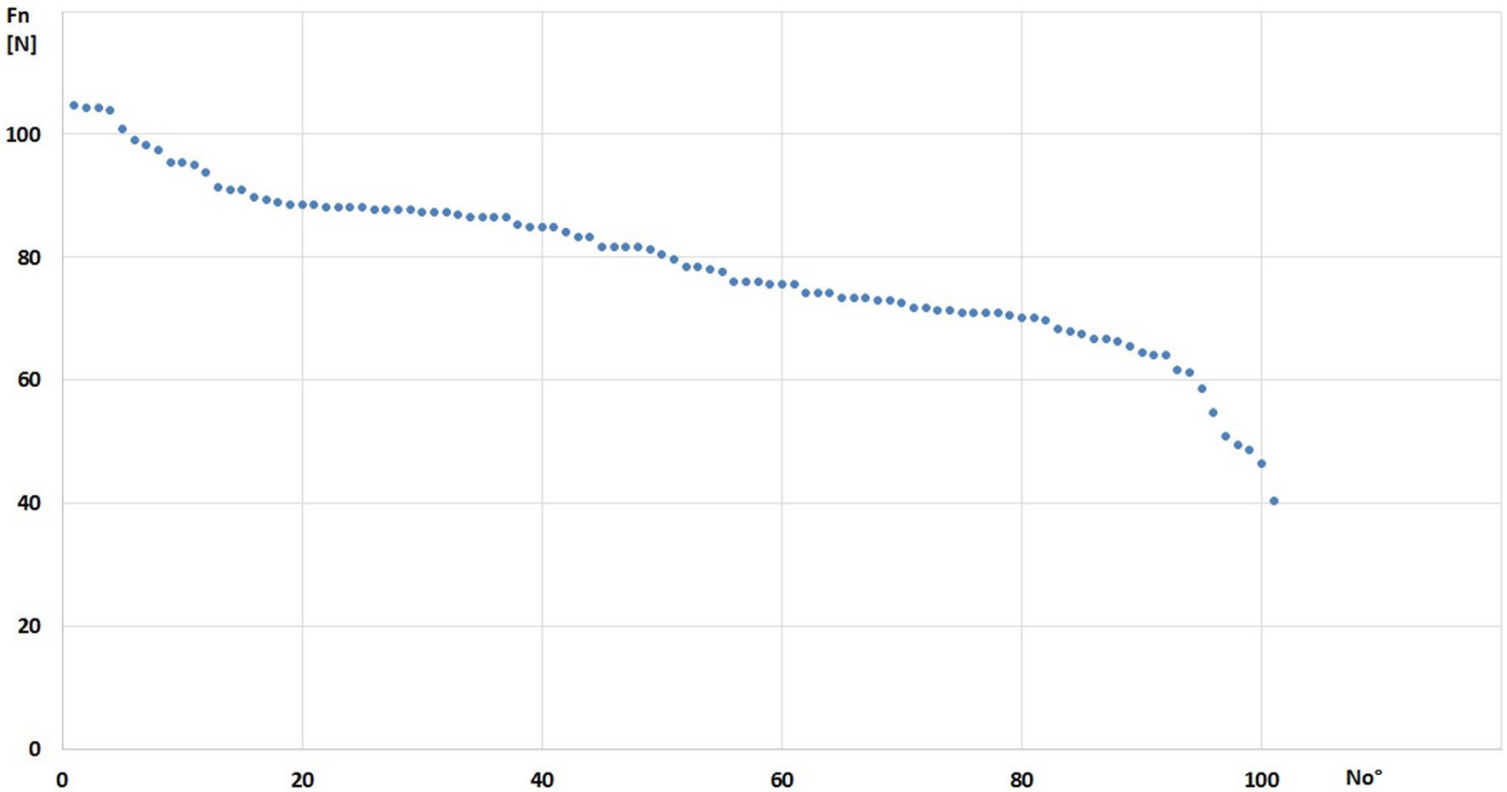
The graph of the distribution of force Fn in the studied group of 101 scapulae

The sample size of 101 scapulae provided > 90% power for all comparisons. The effect size of the differences was large for all comparisons (*d* > 0.8). Table 2 presents data of four independent measurements of SSSA, and Table 3 contains the extended statistical analysis of comparison between these measurements (interclass correlation coefficient). The mean SSSA value was 44.57° (28.5°–67.25° ± 7.9). The most common type of suprascapular notch was Type III (Table 4). No statistically significant correlation was found between notch type and SSSA value (Weka 3 analysis) (Table 4). However, it was found that the SSSA value of group A1 was significantly lower than in group A2 (*U* Mann–Whitney test, *p* < 0.000) and an analogous difference was found between groups B1 and b (*p* < 0.000, *U* Mann–Whitney test) (Table 5). The mean calculated force exerted by the infraspinatus (Fn) on the LTSN was 79 N (40.43N–104.81N ± 13.1) with a nearly linear graph (Fig. 5). Moreover, our mathematical simulation revealed significant differences of Fn between groups A1 and A2 (*p* < 0.000, *U* Mann–Whitney test) and between groups B1 and b (*p* < 0.000, *U* Mann–Whitney test) (Table 5). The average difference of Fn was 28.1% between groups A1 and A2 and 31% between groups B1 and B2 (Table 5).

**Table 2:** The SSSA values according to four independent measurements performed by first author (measurements No. 1 and No. 2) and co-author (measurements No. 3 and No. 4)

Number of measurement	Mean value	Minimum value	Maximum value	Standard deviation
No. 1	43.08	25.00	67.00	8.65
No. 2	45.53	26.00	65.00	7.98
No. 3	44.66	25.00	74.00	9.04
No. 4	45.02	25.00	68.00	7.87

**Table 3:** The extended comparison (interclass correlation coefficient—ICC) between 4 measurements of SSSA performed independently by first author (measurements No. 1 and No. 2) and co-author (measurements No. 3 and No. 4)

Measurements	ICC
Intra-observer	
No. 1/ No. 2	0.80
No. 3/ No. 4	0.86
Inter-observer	
No. 1 + No. 2/No. 3 + No. 4	0.88
No. 1/No. 3	0.88
No. 2/No. 4	0.89
No. 1/ No. 4	0.82
No. 2/No. 3	0.81

**Table 4:** Values of SSSA angle (º) against notch type according to Polguj et al. [31]

Notch type	Mean value	Minimum value	Maximum value	Standard deviation	Number of scapulae	% of scapulae (%)
Type I	45.82	32.75	62.0	8.12	14	13.86
Type II	43.72	37.0	50.25	5.4	8	7.92
Type III	44.47	28.75	67.25	7.73	59	58.41
Type IV	52.49	41.0	61.75	7.75	6	5.94
Type V	40.75	28.5	51.5	7.78	14	13.86

**Table 5:** Mean value, range and standard deviation of the SSSA (º) and Fn (Newtons) calculated based on the literature prevalence of ISA in beach volleyball players (group A1, A2) and tennis players (group B1, B2)

Group	Mean	Minimum	Maximum	Standard deviation
A1-SSSA	36.4	28.5	40.0	3.72
A2-SSSA	48.73	40.0	67.25	6.02
B1-SSSA	38.36	28.5	45.0	4.15
B2-SSSA	51.17	45.0	67.25	5.13
A1-Fn	92.4	86.63	104.81	5.9
A2-Fn	72.15	40.43	86.63	10.15
B1-Fn	89.22	78.45	104.81	6.62
B2-Fn	68.1	40.43	78.45	8.75
Group A1—34% of scapula with lowest SSSA; Group A2—the remaining 66% of scapula Group B1—52% of scapula with the lowest SSSA value; Group B2—the remaining 48% of scapula				

## Discussion

The most important finding of our study is the disclosure of a potential relationship between the anatomical course of LTSN, expressed as SSSA and the aetiology of ISA in throwing sports. Neurogenic ISA affects between 4.4 and 52% of baseball, volleyball and female tennis players [4, 17, 22, 46, 50].

This high frequency of occurrence and the fact that the condition is not found in all players are not accounted for by the proposed pathomechanisms of ISA, in which a crucial role is played by hyper-abduction [4, 12, 17, 21–23, 28, 34, 36, 39, 42, 46, 47, 50], occasionally combined with specific scapular kinematics and increase of shoulder range of motion [47], rare anatomical variants [5, 7, 34, 39, 48] and the presence of cyst in the spinoglenoid notch [20, 45]. Therefore, assuming that a relationship exists between suprascapular nerve neuropathy and damage to the rotator cuff [2, 3, 25, 26, 40], there is a pressing need for more thorough analyses of the biomechanical conditions of LTSN function. No significant correlation was found between SSSA and notch type which was classified according to Polguj et al. [31] (Table 4). Furthermore, the frequency of occurrence of particular notch types was found to be generally in line with those identified in related publications [31–33]. Therefore, we assumed that it was suitable for our hypothetical simulation, which revealed significant differences between group A1 and A2, as well as between group B1 and B2 with regard to SSSA and Fn values.

Collectively, these findings indicate that the development of ISA in different throwing sports may depend on the SSSA value, but the frequency of occurrence is related to existing specific global biomechanical differences between the functional demands placed on the shoulder girdle. Volleyball players are known to complete at least 1000 serves per week [17], which may correspond to 40,000 overhead movements per year, as reported by Dubotzki et al. [6]. Furthermore, according to our mathematical simulation, the hypothetical cumulative mean Fn acting on the LTSN during the year (i.e., the number of serves and spikes × 79 N) would be 3,160,000 N. These figures are clearly unbelievable, and in the case of Group A1, even more so (Table 5). From a pathophysiological perspective, ISA is one of the group of chronic nerve compression injuries of the upper extremities which arises in response to intermittent chronic compression, leading to disturbances in the blood supply to the LTSN [35, 43]. During the early stage of ISA development, compression of the LTSN vessels leads to temporary epineural ischemia [35] which, in the case of volleyball, may be manifested as pathological EMG findings [22]. The chronicity of ISA leads to fibrosis, demyelination and LTSN fibre degeneration [17, 22, 35]. This approach has a strong scientific base, since pathophysiology of chronic nerve compression injuries is independent of axonal damage [35]. Taking these findings together with ours, we propose a new term for isolated infraspinatus atrophy: in our view, the term neuropathy of the lateral trunk of the suprascapular nerve (LTSNN) is more appropriate as it defines the source of the pathology rather than simply describes the clinical symptom.

**Fig. 6:**
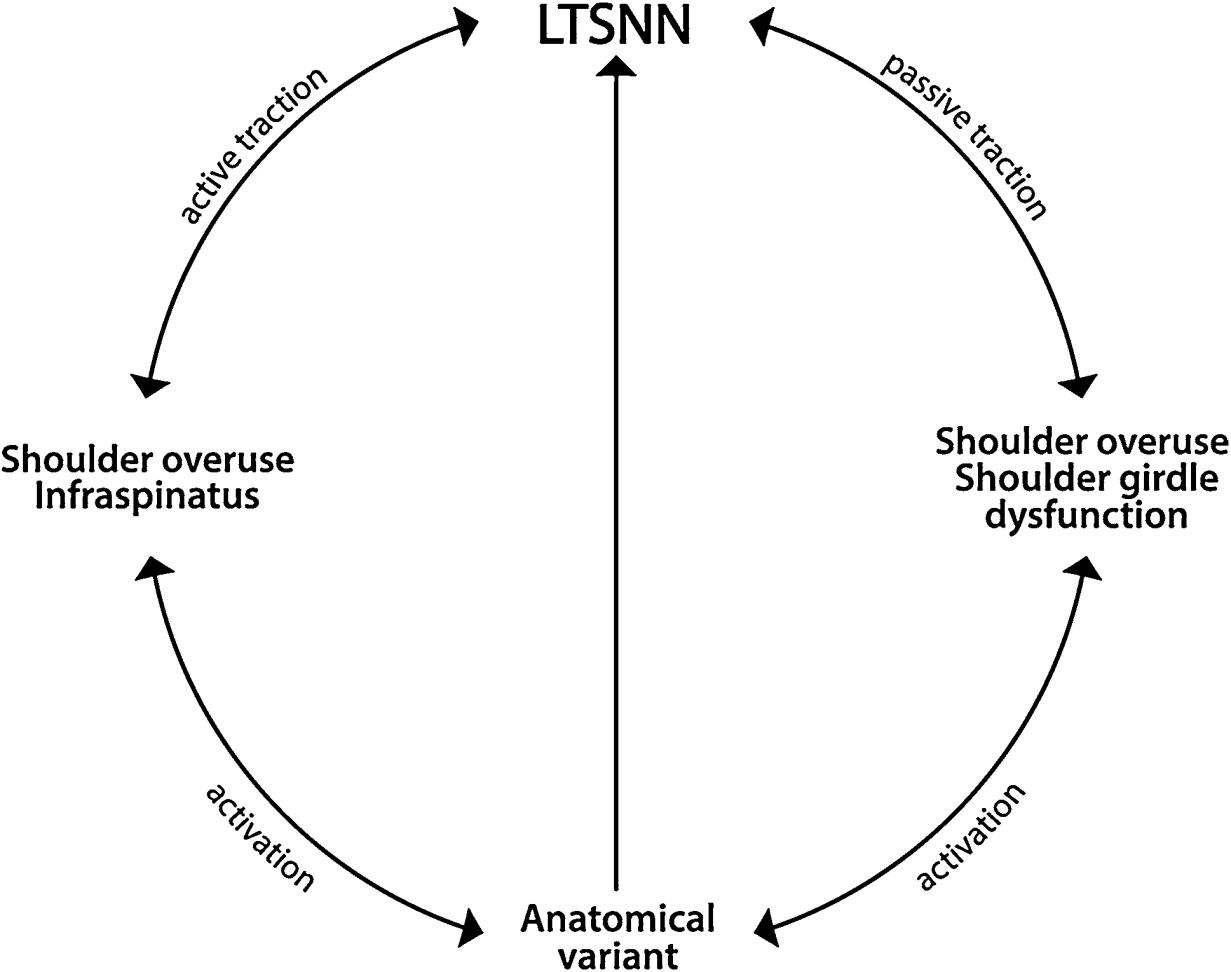
The global biomechanical concept of the development of lateral trunk of suprascapular nerve neuropathy (LTSNN)

In case of throwing sports, Fn acts both as a concentric accelerating force during the windup and cocking phase of shoulder hyper-abduction, and as a decelerating eccentric force immediately afterwards [37, 38]. Therefore, Fn prolongs intermittent disturbances of blood circulation within the LTSN vessels during the specific phases of throwing in spite of the presence of some differences in infraspinatus EMG activity characteristic of a particular throwing discipline [37, 38]. It seems that the Fn exerted by the infraspinatus on LTSN during the decelerating stage is more dangerous than that exerted during acceleration, since it is associated with an increase in the passive stretch of the LTSN due to separation of the neck and scapula, however, this issue requires further study. Moreover, it should be stressed that some other co-existing mechanisms are possible: i.e., excessive range of motion of the shoulder girdle [47], specific scapular kinematics [18, 47] and their relationship to proprioception disturbance [10]. Our global biomechanical concept of LTSNN (ISA) development is summarised in Fig. 6.

The early diagnosis and treatment of LTSNN (ISA) is especially important because only partial recovery of isokinetic peak torque is possible in the external rotators following arthroscopic decompression of the suprascapular nerve [11], as is the case with external rotator strength after open spinoglenoid notch plasty [39] and its decompression [24]. In the light of this pathophysiology [35], 5 the incomplete recovery of muscle atrophy [12] and external rotator strength [24, 39] indicates that the degeneration of some axons associated with an advanced stage of LTSNN development is irreversible. Furthermore, since the recovery of external rotator strength observed following proper training is also connected with hypertrophy of the teres minor [19, 49] the extent of irreversible degeneration of the LTSN axons may be more advanced than first thought.

As LTSNN does not induce any significant clinical shoulder dysfunction and the athlete may continue competing, the clinical significance of this condition is often overlooked [12, 17, 22]. Nevertheless, a wealth of epidemiological data exists concerning rotator cuff pathology and its prevalence is known to increase with age, from 9.7% in patients aged from ≤ 20 years to 62% in those aged ≥ 80 years [44].

Furthermore, atrophy and fatty infiltration of the supraspinatus is known to reach an irreversible stage at an average of 5 years after the onset of symptoms [27]. Taking these findings collectively, irreversible LTSN axon damage associated with rotator cuff tear is a poor prognostic clinical finding during a sporting career, and even more so after its completion [2]. Recent experimental [9, 30] and clinical data indicates that muscle atrophy is reversible following successful reconstruction of the rotator cuff; however, restoration of the infraspinatus will not be possible if accompanied by the irreversible stage of the LTSNN. The study has some limitations. The evaluation of the LTSN course may be subject to some error although it should be stressed that the method of SSSA measurement is based on stable bone landmarks determined at 18 formalin-fixed cadaveric shoulders and the extended evaluation of interclass correlation coefficient proved its reliability. In addition, the age, sex and range of differences side to side of the examined scapulae were not known. However, according to Paraskevas et al. [29] the shape of the acromion was symmetrical in 65.9% of cases, and their results reveal no correlation between shape and gender. Shi et al. [41] confirmed that there is excellent side-to-side symmetry in glenoid shape regardless of sex. Moreover, Polguj et al. [32] note that the distribution of the suprascapular notch types is similar between the two sexes. Sexual dimorphism was further investigated by Polguj et al. [33] on total of 616 computer tomography scans of shoulders randomly selected from the database of patients who were being investigated due to chest problems. This study revealed that the frequency of type I and IV was lower in females than in males, but type III was more common in females than males. The other types of suprascapular notch had similar distributions in the two sexes. Additionally, the authors revealed that from among three dimensions characterising the type of notch only the maximal notch depth was statistically significantly higher in males than in females [33]. In contrast to Polguj et al. [33], Albino et al. [1] reported no correlation between notch type and gender, as well as age. This study was done on 500 dry scapulae. However, it should be noted that the Albino et al. [1] study applied other than Polguj et al. [33] notch classification.

Furthermore, Gumina et al. [14] examined 500 dried scapulae, measuring six distances for each one (referring to the scapular body, glenoid, and the course of the suprascapular nerve). They found insignificant differences between the right and left scapulae, and significant differences between those taken from males and females. Therefore, based on all these findings, together with the lack of literature about the impact of correlation between the sex and particular parameters of the scapula on isolated infraspinatus atrophy prevalence in throwing sports it is seemed that age, sex and side have no important impact on the SSSA measurement and value. In addition, the contraction of the supraspinatus may also temporarily affect the course of the LTSN leading to the decrease of SSSA value [30], which was not taken into account. The mean value of the infraspinatus force F was related to its selective activity in common shoulder rehabilitation exercises [8]; despite this, the key issue is the SSSA value which finally influences the magnitude of Fn.

Nevertheless, despite these limitations, our findings represent an important contribution which deepens the understanding of the pathomechanism of LTSNN development in throwing sports. Moreover, it serves as a basis for determining MRI and CT protocols for SSSA evaluation, as well as for further clinical studies intended to highlight the method of selecting athletes at risk of LTSNN development, and its subsequent treatment in the case of occurrence.

1. The course of the lateral trunk of the suprascapular nerve, defined as the suprascapular-scapular angle, is a feature specific to an individual.
2. The results of the study revealed the potential relationship between the anatomical course of the lateral trunk of the suprascapular nerve expressed as the suprascapular-scapular angle and the aetiology of isolated infraspinatus atrophy development.

## Abbreviations

ISA: Isolated infraspinatus atrophy
LTSN: Lateral trunk of the suprascapular nerve
SSSA: Suprascapular-scapular spine angle
Fn: Resultant compressive force
*F*: Pulling force of insfraspinatus contracture
